# The effects of size and period of administration of gold nanoparticles on rheological parameters of blood plasma of rats over a wide range of shear rates: In vivo

**DOI:** 10.1186/1476-511X-10-191

**Published:** 2011-10-27

**Authors:** Mohamed Anwar K Abdelhalim

**Affiliations:** 1 Physics and Astronomy, King Saud University, College of Science, Riyadh-11451, P.O. 2455, Saudi Arabia

**Keywords:** Gold nanoparticle, sizes, blood plasma, periods, rheological parameters, rats

## Abstract

**Background:**

Blood viscosity appears to be independent predictor of stroke, carotid intima-media thickening, atherosclerosis and most cardiovascular diseases. In an attempt to understand the toxicity and the potential threat of GNPs therapeutic and diagnostic use, an array of rheological parameters were performed to quantify the blood plasma response to different sizes and administration periods of GNPs over a wide range of shear rates.

**Methods:**

Healthy, thirty male Wistar-Kyoto rats, 8-12 weeks old (approximately 250 g body weight) were divided into control group (NG: n = 10), group 1 (G1A: intraperitoneal infusion of 10 nm GNPs for 3 days, n = 5 and G1B: intraperitoneal infusion of 10 nm GNPs for 7 days, n = 5), group 2 (G2A: intraperitoneal infusion of 50 nm GNPs for 3 days, n = 5 and G2B: intraperitoneal infusion of 50 nm GNPs for 7 days, n = 5). Dose of 100 μl of GNPs was administered to the animals via intraperitoneal injection. Blood samples of nearly 1 ml were obtained from each rat. Various rheological parameters such as torque, shear stress, shear rate, viscosity, plastic velocity, yield stress, consistency index (k) and flow index (n) were measured in the blood plasma of rats after the intraperitoneal administration of 10 and 50 nm GNP for 3 and 7 days using Brookfield LVDV-III Programmable rheometer.

**Results:**

The relationship between shear stress and shear rate for control, G1A, G1B, G2A and G2B was linearly related. The plastic viscosity and the yield stress values for G1A, G1B, G2A and G2B significantly (p < 0.05) decreased compared with the control. The n and k values calculated from equation (1). The k values for G1A, G1B and G2B decreased compared with the control; however the means were not significantly different. While G2A indicates no significant change compared with the control. The values of the flow behaviour index (n) were equal ≤ 1 for all the different GNPs sizes. The viscosity values measured for 10 and 50 nm GNPs (G1A, G1B, G2A and G2B) decreased compared with the control; however the means were not significantly different. The decrease in blood plasma viscosity values observed with all GNPs is particle size and administration period independent.

**Conclusions:**

At these particular shear rates, the estimated rheological parameters are not influenced by GNPs size and shape, number of NPs, surface area and administration period of GNPs. This study demonstrates that the highly decrease in blood plasma viscosity was accompanied with the smaller 10 nm GNPs compared with the 50 nm GNPs. The decrease in blood plasma viscosity induced with 10 and 50 nm GNPs may be attributed to decrease in hematocrit and haemoglobin concentration in addition to erythrocyte deformability. This study suggests that histomorphologcal, histochemical and ultrastrucural investigations are needed to evaluate the inflammations and tissue injuries, in relation to the application of GNPs as a therapeutic and diagnostic tool.

## Introduction

The elevation of whole-blood viscosity is a predictor of stroke, carotid intima-media thickening, and atherosclerosis. However, in most studies, whole blood viscosity was measured at a few non-specific shear rates and these data not reflect the complete rheological characteristics in these studies [1-3].

Erythrocytes change their shape extensively under the influence of applied forces in fluid flow or while passing through microcirculation. The extent and geometry of this shape change is determined by both the rheological properties of erythrocytes, the magnitude of the applied forces and the orientation of erythrocytes with the applied forces [1].

Blood viscosity is determined by plasma viscosity, hematocrit and rheological behavior of red blood cells. Therefore, red blood cell mechanics is the major determinant of flow properties of blood. Red blood cells have unique rheological behavior, which can be discussed under the terms erythrocyte deformability and erythrocyte aggregation [2].

Whole-blood Blood is a unique fluid, it exhibits non-Newtonian characteristics, and its viscosity is dependent on shear rate. Major determinants of whole-blood viscosity are haematocrit, plasma viscosity, and red cell aggregation and red cell deformation under conditions of low and high shear rates [4-6].

Plasma fibrinogen concentration and plasma viscosity are elevated in unstable angina pectoris and stroke and their higher values are associated with higher rate of major adverse clinical events. Elevation of plasma viscosity correlates to the progression of coronary and peripheral artery diseases. Plasma viscosity should be measured routinely in medical practice [7].

A study on nanoparticle is becoming a hot point owing to their novel physical and chemical attributes in biology and medicine [8-15].

Owing to the unique optoelectronic properties with their controlled size and morphology [8], GNPs find significance in the field of bionanotechnology [8] as biomarkers [11], cancer diagnostic [8,15], as photo-thermal agents in hyperthermia [14], gene expression [12], and DNA detection [10].

The size of GNPs is similar to that of most biological molecules; therefore, GNPs can be useful for both in vivo and in vitro biomedical research and applications. Thus, an increased attention is focused on the applications of nanoparticles in biology and medicine.

The effect of storage time and temperature on the quality of GNPs as pharmaceutical products can be investigated via rheological measurements. Rheological analysis can be employed as a sensitive tool in predicting the physical properties of the different GNP sizes [16].

The objective of this study was to quantify array of rheological parameters for the blood plasma of rats to different sizes and periods administration of GNPs over a wide range of shear rates.

## Materials and methods

### Gold nanoparticles size

10 and 50 nm GNPs were purchased (Product MKN-Au, Canada) and used in this study. The 10 nm GNPs were dissolved in aqueous solution (Product MKN-Au-010; concentration 0.01% Au) and 50 nm GNPs (Product MKN-Au-050; concentration 0.01% Au).

### Animals

Healthy, male Wistar-Kyoto rats were obtained from the Laboratory Animal Centre (College of Pharmacy, King Saud University). 8-12 weeks old (approximately 250 g body weight) were housed in pairs in humidity and temperature-controlled ventilated cages on a 12 h day/night cycle. A rodent diet and water were provided. In this study, thirty rats were individually caged, and divided into control group (NG: n = 10), group 1 (G1A: infusion of 10 nm GNPs for 3 days, n = 5 and G1B: infusion of 10 nm GNPs for 7 days, n = 5), group 2 (G2A: infusion of 50 nm GNPs for 3 days, n = 5 and G2B: infusion of 50 nm GNPs for 7 days, n = 5). Dose of 100 μl of 10 and 50 nm GNPs dissolved in aqueous solutions were intraperitonealy administered daily to the animals for periods of 3 and 7 days. The rats were anesthetized by inhalation of 5% isoflurane until muscular tonus relaxed. Blood samples of nearly 1 ml were obtained from each rat. 1 ml of Blood was collected into polypropylene tubes viz., the blood for plasma was collected in EDTA. All experiments were conducted in accordance with the guidelines approved by King Saud University Local Animal Care and Use Committee.

### Experimental set up and rheological parameters measurement

Several rheological parameters for the blood plasma of rats after the administration of 10 and 50 nm GNPs for periods of 3 and 7 days were measured. The rheological parameters were viscosity, torque, shear stress, shear rate, plastic viscosity, yield stress, consistency index and flow index. These rheological parameters were measured using Brookfield LVDV-III Programmable rheometer (cone-plate viscometer; Brookfield Engineering Laboratory, Incorporation, Middleboro, USA) supplied with temperature bath controlled by a computer. The rheometer was guaranteed to be accurate within ± 1% of the full scale range of the spindle/speed combination in use reproducibility is within ± .2%.

Rheological parameters were measured at temperature of 37°C. Temperature inside the sample chamber was carefully monitored using a temperature sensor during the rheological parameters measurement.

A cone and plate sensor having a diameter of 2.4 cm with an angle of 0.8 was used. The rheometer was calibrated using the standard fluids. This viscometer has a viscosity measurement range of 1.5-30,000 mPas.

The spindle type (SC-40) and its speed combinations will produce results with high accuracy when the applied torque is in the range of 10% to 100% and accordingly the spindle is chosen.

0.5 ml of each GNP size dissolved in aqueous solution was poured in the sample chamber of the rheometer. The spindle was immersed and rotated in these gold nanofluids in the speed range from 20 to 180 RPM in steps of 20 minutes. The viscous drag of the GNP aqueous solution against the spindle was measured by the deflection of the calibrated spring.

### Plastic viscosity and yield stress

The flow curves were plotted between shear stress (dyne/cm^2^) and shear rate (s^-1^) for each GNP size. Plastic viscosity and yield stress were calculated from the linear fitting of the flow curves [16,17].

### Consistency index (k) and flow index (n)

The consistency index and flow index for the 10 and 50 nm GNPs in aqueous solution can be evaluated by power law model [17]:

(1) $$\tau =k\nu^{n}$$

Where τ is the shear stress, k is the consistency index, ν is the shear rate, and n is the flow behaviour index [17].

### Statistical analysis

Results of this study were expressed as Mean ± standard deviation (Mean ± SD). The significance of difference between the control and the other groups (G1A and G1B, and G2A and G2B) was performed using one-way analysis of variance (ANOVA) for repeated measurements, with significance assessed at 5% confidence level.

## Results and discussions

### Rheological parameters measurement

**Figure 1:**
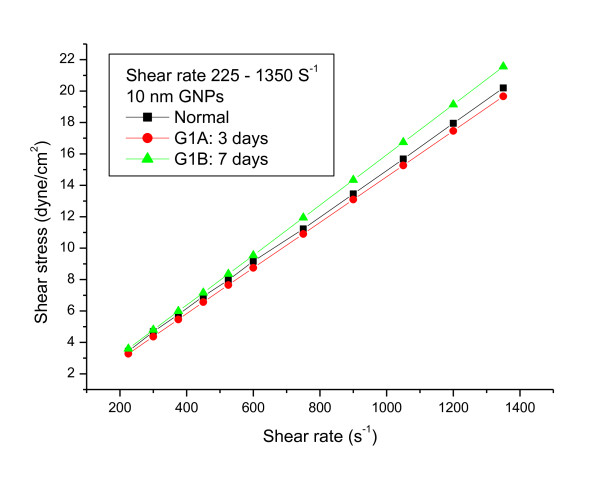
**The relationship between shear stress and shear rate for blood plasma of rats with 10 nm gold nanoparticles at periods of 3 and 7 days (the means are not significantly different (p < 0.05))**.

**Figure 2:**
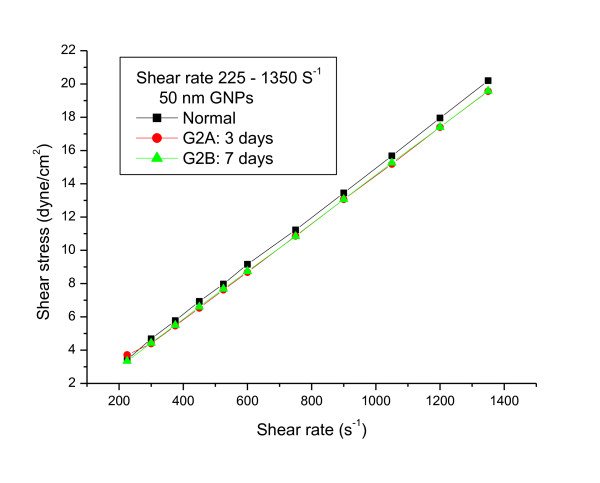
**The relationship between shear stress and shear rate for blood plasma of rats with 50 nm gold nanoparticles at periods of 3 and 7 days (the means are not significantly different (p < 0.05))**.

**Figure 3:**
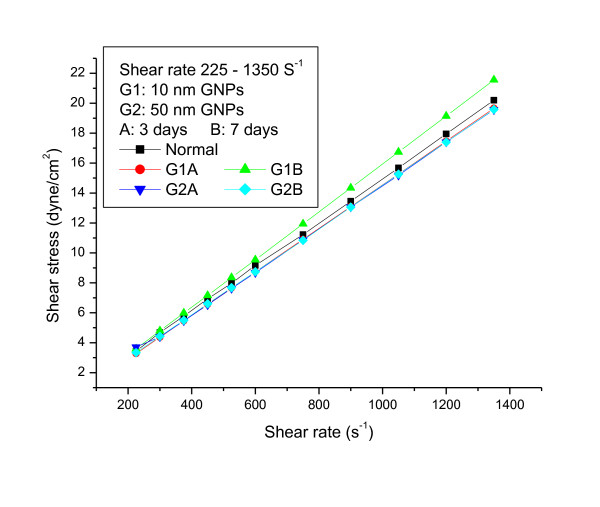
**The relationship between shear stress and shear rate for blood plasma of rats with 10 and 50 nm gold nanoparticles at periods of 3 and 7 days (the means are not significantly different (p < 0.05))**.

The relationship between shear stress and shear rate for 10 and 50 nm GNPs administered daily into rats for periods of 3 and 7 days at wide range of shear rates (from 225 to 1350s^-1) ^and at fixed temperature of 37°C were measured (Figures 1, 2 and 3).

The relationship between shear stress and shear rate was linearly related. The linear flow rate relation for the 10 and 50 nm GNPs were described by the following equations:

$$\mathrm{Control}:\;\text{Y }=\;0.0\text{148 X }+\;0.\text{21}0\text{1 }\left(\text{R }=\;0.\text{999}\right)$$

$$10\mathrm{nm}\left(G1A\right):\;\text{Y }=\;0.0\text{146 X }+\;0.00\text{6}0\;\left(\text{R }=\text{ 1}.000\right)$$

$$10\mathrm{nm}\left(G1B\right):\;\text{Y }=\;0.0\text{159 X }-0.0\text{138 }\left(\text{R }=\;0.\text{999}\right)$$

$$50\mathrm{nm}\left(G2A\right):\;\text{Y }=\;0.0\text{143 X }+\;0.\text{1691 }\left(\text{R }=\;0.\text{999}\right)$$

$$50\mathrm{nm}\left(G2B\right):\;\text{Y }=\;0.0\text{144 X }+\;0.0\text{9}0\text{6 }\left(\text{R }=\;0.\text{999}\right)$$

The plastic viscosity (a measure of the internal resistance to fluid flow expressed as the tangential shear stress in excess of the yield stress divided by the resulting rate of shear)[17], yield stress (dyne/cm^2^) (the minimum stress needed to cause the flow), consistency index (k) (an indication of the viscous nature of GNPs), the flow behaviour index (n) (a measure of departure from Newtonian flow) were calculated for different GNP sizes as shown in Additional File - Table 1.

### Rheological parameters measurement for blood plasma of rats with different sizes and periods of administration of GNPs (Additional File 1, Table 1)

The plastic viscosity and yield stress parameters were calculated from fitting the experimental data for the different GNPs sizes. The plastic viscosity and the yield stress values for G1A, G1B, G2A and G2B significantly (p < 0.05) decreased compared with the control.

The n and k values were calculated from equation (1). The k values for G1A, G1B and G2B decreased compared with the control; however the means were not significantly different. While G2A indicates no significant change compared with the control. The values of the flow behaviour index (n) were equal ≤ 1 for all the different GNPs sizes.

The viscosity values measured for the different GNPs sizes at periods of 3 and 7 days and wide range of shear rate (375-1875s^-1^) are shown in Additional File 1, Table 1. The viscosity values for 10 and 50 nm GNPs (G1A, G1B, G2A and G2B) decreased compared with the control; however the means were not significantly different. The decrease in blood plasma viscosity observed for all GNPs is particle size and administration period independent.

The data in the present study suggests that, at these particular shear rates, the estimated rheological parameters are not influenced by particles size, shape, number of NPs, surface area and administration period of GNPs.

This study suggests that the decrease in blood plasma viscosity induced with 10 and 50 nm GNPs may be attributed to decrease in hematocrit and cytoplasmic haemoglobin concentration of erythrocytes, and to the high erythrocyte deformability (the degree of shape change under a given level of applied shear stress and shear rate). The higher temperature or pH sensitivity of protein has higher plasma viscosity results. Inflammations, tissue injuries resulting in plasma protein changes can increase its value with high sensitivity, though low specificity [6,7,17].

The factors that affect blood viscosity are plasma protein concentration and types of proteins in plasma, but these effects are so much less than the effect of hematocrit. The elevation of plasma viscosity correlates to the progression of coronary and peripheral vascular diseases. Anaemia can lead to decrease in blood viscosity, which may lead to heart failure [1-3].

The deformability is an intrinsic property of erythrocytes which can be determined by the geometric and material properties of this unique cell [6]. Erythrocytes change their shape extensively under the influence of applied shear stress in fluid flow or while passing through microcirculation. The extent and geometry of this shape change is determined by the rheological properties of erythrocytes, the magnitude of the applied forces and the orientation of erythrocytes with the applied forces.

The viscoelastic behaviour of erythrocytes is determined by the following three properties: 1) Geometry of erythrocytes; the biconcave-discoid shape provides an extra surface area for the cell, enabling shape change without increasing surface area. 2) Cytoplasmic viscosity; reflecting the cytoplasmic haemoglobin concentration of erythrocytes. 3) Visco-elastic properties of erythrocyte membrane, which can mainly be determined by the special membrane skeletal network of erythrocytes [5,17].

The effect of protein on plasma viscosity depends on its molecular weight and structure. The less spheroid shape is the higher molecular weight and aggregating capacity. The higher temperature or pH sensitivity of protein has the higher plasma viscosity results [4,7].

Human blood plasma is a Newtonian fluid and its viscosity does not depend on flow characteristics, its control value is 1.10-1.30 mPas at 37°C, and it does not depend on the age and gender [6].

The GNPs strongly associate with the essential blood proteins where the binding constant as well as the degree of cooperativity of particle-protein binding, depends on particle size and the native protein structure. Moreover, the thickness of the adsorbed protein layer progressively increases with NP size, effects that have potential general importance for understanding NP aggregation in biological media and the interaction of NP with biological materials [9,11,18,19].

This study suggests that the administration of GNPs may induce appearance of hepatocytes cytoplasmic degeneration and nuclear destruction due to the interaction with proteins and enzymes of the hepatic tissue, which in turn interfering with the antioxidant defence mechanism and leading to reactive oxygen species (ROS) generation which induce stress in the hepatocytes to undergo atrophy and necrosis. Thus, to evaluate inflammations and tissue injuries, more histomorphologcal, histochemical and ultrastrucural investigations are needed in relation to the application of GNPs with their potential threat as a therapeutic and diagnostic tool.

## Conclusions

This study is unique in evaluating various rheological parameters for blood plasma of rats after intraperitoneal administration of 100 μl of 10 and 50 nm GNPs for periods of 3 and 7 days at fixed temperature of 37°C and wide range of shear rates using Brookfield LVDV-III Programmable rheometer.

The relationship between shear stress and shear rate observed with 10 and 50 nm GNPs confirmed with the power law behaviour.

The mean viscosity values measured for 10 and 50 nm GNPs (G1A, G1B, G2A and G2B) decreased compared with the control; however the means were not significantly different. The decrease in blood plasma viscosity values observed with all GNPs is particle size and administration period independent. The decrease in blood plasma viscosity may be attributed to decrease in hematocrit and cytoplasmic haemoglobin concentration of erythrocytes, and to the high erythrocyte deformability. The decrease in blood viscosity may lead to anaemia and/or heart failure.

The data in the present study demonstrates that, at these particular shear rates, the estimated rheological parameters are not influenced by particle size, shape, number of NPs, surface area and administration period of GNPs.

This study suggests that the intraperitoneal administration of GNPs may induce appearance of hepatocytes cytoplasmic degeneration and nuclear destruction due to the interaction with proteins and enzymes of the hepatic tissue, which in turn interfering with the antioxidant defence mechanism and leading to reactive oxygen species (ROS) generation.

Thus, to evaluate inflammations and tissue injuries associated with GNPs, more histomorphologcal, histochemical and ultrastrucural investigations are needed in relation to the application of GNPs as a therapeutic and diagnostic tool.

## Competing interests

The author declares that he has no competing interests.

## Authors' contributions

AMAK has analyzed data, interpreted and written the final draft of this manuscript. The animal model used in this study was obtained from the Laboratory Animal Center (College of Pharmacy, King Saud University). AMAK has conceived the plan and the design, and has obtained the research grant for this study. Moreover, the author has read and approved the final manuscript.

## Supplementary Material

Additional file 1 **Table 1**. Rheological parameters measurement for blood plasma of rats with different sizes and periods of administration of GNPs. Click here for file
