# Supplementary material for: The effects of size and period of administration of gold nanoparticles on rheological parameters of blood plasma of rats over a wide range of shear rates: In vivo
Source: Lipids Health Dis. 2011 Oct 27;10:191. doi: 10.1186/1476-511X-10-191 (PMC3305902; doi:10.1186/1476-511X-10-191)
Supplement: Additional file 1 — Table 1. Rheological parameters measurement for blood plasma of rats with different sizes and periods of administration of GNPs. [file 1476-511X-10-191-S1.DOCX]

| Sample Control G1A G1B G2A G2B  Blood plasma 10 nm GNPs 10 nm GNPs 50 nm GNPs 50 nm GNPs  3 days 7 days 3 days 7 days |
| --- |
| Plastic 1.478± 0.003 1.460± 0.013^*^ 1.445± 0.024^*^ 1.430± 0.015^*^ 1.440 ± 0.012^*^  viscosity (cP) |
| Yield stress 0.210 +0.012 0.006+0.014^*^ 0.014+0.013^*^ 0.169 ± 0.002^*^ 0.091 ± 0.001^*^  (dyne/cm^2^) |
| Consistency 0.018+0.014 0.015+0.016 0.016+0.012 0.019+0.011 0.016+0.021  index (cP) (k) |
| Flow index (n) 0.973 0.997 0.997 0.957 0.985 |
| Viscosity (cP) 1.517+0.025 1.457+0.003 1.453+0.002 1.451+0.006 1.459+0.012 |

*****p<0.05
